# Public Health Workforce Development Through Virtually Interactive Training Courses

**DOI:** 10.3389/phrs.2022.1604657

**Published:** 2022-03-16

**Authors:** Salman Rawaf, Celine Tabche

**Affiliations:** WHO Collaborating Centre for Public Health Education and Training Imperial College London, London, United Kingdom

**Keywords:** public health workforce development, public health training courses, public health education, virtual interactive training courses, workforce development, transformative public health education

## Introduction

One of the main lessons of the COVID-19 pandemic that can be drawn is that the health professional’s education system needs significant revitalisation, especially public health [1]. Public health education and training need a more comprehensive and unique approach to equip its learner’s key concepts through the competencies required for each level of public health practice [2]. In the fourth industrial revolution era, public health professionals need to be part of a new generation of professionals equipped with new skills and appropriate attributes to understand better and serve the population [3]. We developed some bespoke virtual interactive courses that are crucial in training the public health workforce over the past 12 months. These training courses are tailored according to the target audience and the corresponding competencies needed to fill a noticeable gap in the field [4].

## Public Health Workforce

In addressing the above aim, we looked at the ten public health functions and how we can link a set of competencies for each function and assess each competency’s suitability for the target group. Then, the number of competencies needed for each course was identified through a linkage process to the learning objectives. We reached to as many possible stakeholders who identified their selves as involved in public health delivery directly or indirectly, guided by the Public Health England scoping exercise on the public health workforce [5].

We identified the need for an appropriate learning model to be chosen for each course. A learning model, in this context, is a structured content of learning materials presented together. It supports a course concept or theme, goal, learning objectives and topic(s). The essential components of a learning model are the analysis of the situational factors [6]. This gave us information about the target trainees and the expectations from the organisation, formulating the learning goals and objectives of a course that allows the course leader and trainee to evaluate the progress throughout the sessions.

The key element is the selection of the training activities that help choose the appropriate methods of training depending on the content of the course given. While a course leader sets the scope, learners can negotiate a learning method to suit their learning styles and explore the contents in any order and at their own pace. A model that serves its purpose successfully has been developed around the needs of the trainees. These innovative approaches were well received by the trainees.

## Target Groups

Three different potential target groups of the public health workforce were identified. First group is the “Wider Public Health Workforce”: An individual who is not a practitioner or specialist in public health but can positively influence health and wellbeing through their work. This workforce group comprises individuals across a range of sectors, within and outside the health sector. Second group is the “Public Health Practitioners”: They are the core public health workforce who work in various areas of public health, including health improvement, protection, and public health healthcare. Also, in public (service and academic), private, voluntary, and community sectors that contribute to public health outcomes and improve health and wellbeing. Third group is the “Specialists (Consultants) in Public Health” the highest level of the public health workforce. Those described as specialists are medical and non-medical graduates who completed their higher specialists training in public health or preventive medicine for 4–5 years in a well-defined and structured training programme.

This classification of the public health workforce helped design the courses, target audiences, and competencies needed to bridge the gaps through these innovative training methods.

## Phasing out

A “Phase Out” is to denote the actions taken to equip those who are currently employed within public health delivery without public health background, should obtain the accredited training in public health to deliver services based on the science and art of promoting health, preventing disease, and prolonging life through the organised efforts of society [7].

**FIGURE 1:**
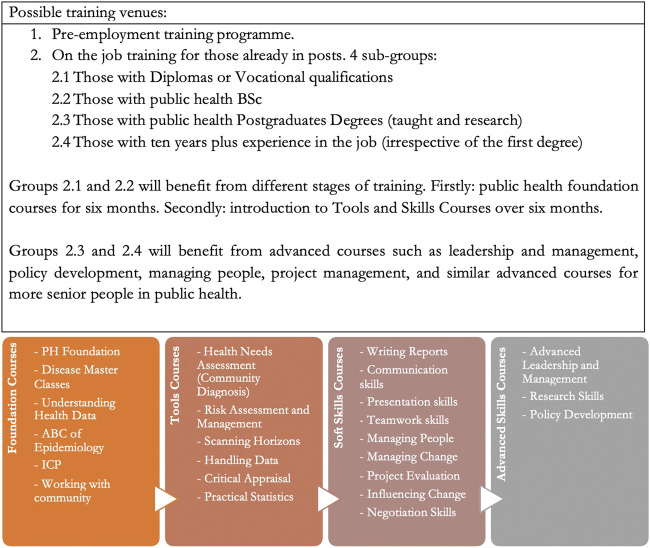
Table explaining the different venues for public health workforce training and a list of different types of public health training courses (United Kingdom, 2020).

All stakeholders involved in public health delivery, directly or indirectly, would welcome such an opportunity to improve the quality of their workforce. This could be achieved through various venues, as outlined in Figure 1. The phase-out process must be guided by a “national public health workforce strategy”. Such a strategy should guide how the future public health workforce’s needs are addressed.

## Bridging the Training Gaps

Based on the ten public health functions, the competencies needed, and gaps identified in the training of groups below consultant level, i.e., those who did not complete a structured training programme over 4–5 years, four types of training courses were identified (Figure 1). Public health foundation courses are designed for those who did not have any education in public health; public health tools courses for all three levels; soft skills courses for all; and advanced skills courses for practitioners and specialists (consultants). These two groups could benefit from joint bespoke design and delivery such as data management, research, and development, policy based on evidence, and health need and impact assessment.

Each course planned for delivery should define the competencies that the course will address. The number of competencies needed for each course must be linked to the learning objectives of the course and the target group.

Candidates joining the training courses should accumulate the most significant number of relevant competencies and be assessed accordingly. It is expected that those aspiring to “specialist” (level 3) should meet 100% of the competencies listed; those for level 2 should achieve 70–75% of the competencies, and those in level 1 (the wider public health workforce) should earn 50% of the competencies. Each candidate will have an electronic logbook to bank the competencies achieved and score the level reached.

### Conclusion

We started these courses virtually; however, this framework should continue into hybrid and physical teaching. This systematic way of identifying the needed skills and what is missing from the public health workforce should be used for future public health education and training courses to develop the appropriate learning platform to fulfil the needed competencies and requirements for a well-functioning and full accredited public health system the people can trust.
